# Protein engineering of conger eel galectins by tracing of molecular evolution using probable ancestral mutants

**DOI:** 10.1186/1471-2148-10-43

**Published:** 2010-02-14

**Authors:** Ayumu Konno, Shintarou Yonemaru, Atsushi Kitagawa, Koji Muramoto, Tsuyoshi Shirai, Tomohisa Ogawa

**Affiliations:** 1 Department of Biomolecular Science, Graduate School of Life Sciences, Tohoku University, Sendai 981-8555, Japan; 2 Nagahama Institute of Bio-Science and Technology and Japan Science and Technology Agency, BioInfomatic Research Division, Nagahama, Shiga 526-0829, Japan

## Abstract

**Background:**

Conger eel galectins, congerin I (ConI) and congerin II (ConII), show the different molecular characteristics resulting from accelerating evolution. We recently reconstructed a probable ancestral form of congerins, Con-anc. It showed properties similar to those of ConII in terms of thermostability and carbohydrate recognition specificity, although it shares a higher sequence similarity with ConI than ConII.

**Results:**

In this study, we have focused on the different amino acid residues between Con-anc and ConI, and have performed the protein engineering of Con-anc through site-directed mutagenesis, followed by the molecular evolution analysis of the mutants. This approach revealed the functional importance of loop structures of congerins: (1) N- and C-terminal and loop 5 regions that are involved in conferring a high thermostability to ConI; (2) loops 3, 5, and 6 that are responsible for stronger binding of ConI to most sugars; and (3) loops 5 and 6, and Thr38 residue in loop 3 contribute the specificity of ConI toward lacto-*N*-fucopentaose-containing sugars.

**Conclusions:**

Thus, this methodology, with tracing of the molecular evolution using ancestral mutants, is a powerful tool for the analysis of not only the molecular evolutionary process, but also the structural elements of a protein responsible for its various functions.

## Background

Molecular evolution refers to the evolutionary process at the macromolecular level, such as at the DNA, RNA, and protein levels. It encompasses the reconstruction of the evolutionary history of organisms and macromolecules (i.e., molecular phylogeny) on the basis of the sequence data of nucleic acids and proteins. The primary event in molecular evolution is a mutational change in genes that may be caused by the substitution or insertion/deletion of a nucleotide, recombination, etc.; otherwise, in general, DNA sequences are copied exactly during the process of chromosome replication. Subsequently, they spread in a population by genetic drift and/or natural selection, and eventually get established in a species [1,2]. Thus, the evolutionary history over a period of multibillion years has its basis in the DNA. To understand the molecular evolution of proteins in nature, we usually refer to the relationships and rates of changes in the sequence data inferred from proteins identified so far. More recent advances in bioinformatics and structural biology, besides recombinant protein expression techniques, have enabled us to analyze the molecular evolution of proteins more directly, explore the evolutionary strategies of natural proteins, and generate novel tailor-made proteins.

Galectins are defined as proteins having at least one characteristic carbohydrate-recognition domain with Ca^2+^-independent affinity for β-galactoside, and they share certain conserved sequence elements [3]. To date, 15 galectins have been identified in mammals. They are involved in many biological phenomena, including cell adhesion, differentiation, morphogenesis, innate immunity, apoptosis, and metastasis of malignant cells [4-8]. Furthermore, the members of the galectin family have been isolated from a large variety of metazoan phyla, from invertebrates such as nematodes, insects, and sponges to vertebrates such as fish and chicken, as well as mammals [9,10]. On the basis of their structures, galectins are classified into three types: proto-, chimera-, and tandem repeat-type galectins [11].

Conger eel (*Conger myriaster*) contains two proto-type galectins, namely, congerin I (ConI) and congerin II (ConII) in the skin mucus [12,13]. ConI and ConII consist of 136 and 135 amino acid residues, respectively, and both contain acetylated N-termini [13,14]. However, they have no cysteine residue that is related to oxidizing inactivation found in some galectins of higher vertebrates. Congerins are considered to participate in the host defense against infectious agents, such as bacteria and parasites. For example, ConI and ConII mainly exist in the frontier organs and tissues that delineate the body from the outer environment, such as the epidermal club cells of the skin, wall of the oral cavity, pharynx, esophagus, and gills; in addition, they also exhibit agglutination activity against the marine pathogen, *Vibrio anguillarum *[12,15,16]. Moreover, it was recently reported that congerins can exert opsonic effects and can reach the intestinal lumen without enzymatic digestion [17,18].

The molecular evolutionary and X-ray crystallography analyses of ConI and ConII revealed that they have evolved in an accelerating manner, resulting in the emergence of new structures, including the strand-swap structure and a unique carbohydrate-binding site; this in turn resulted in a unique carbohydrate-binding ability [15,19-22]. We recently reconstructed a probable ancestral form of congerin (Con-anc) and found that it showed properties similar to those of ConII in terms of thermostability and carbohydrate-recognition specificity, except for α2,3-sialyl galactose, although Con-anc was observed to share a higher sequence similarity with ConI than ConII [23]. This indicates that only the 31 different amino acid residues between ConI and Con-anc are involved in conferring the characteristics of ConI, which are acquired during its adaptive molecular evolution from Con-anc. To identify the determinants of selection pressures in the evolutionary process and the structural elements associated with the unique carbohydrate-binding activities of ConI, in the present study, we focused on the different amino acid residues between Con-anc and ConI, and conducted a dissection analysis using the chimera mutants of Con-anc and ConI, tracing its evolutionary history to ConI.

## Results and Discussion

### Design and preparation of the chimeric mutants of Con-anc and ConI

**Figure 1:**
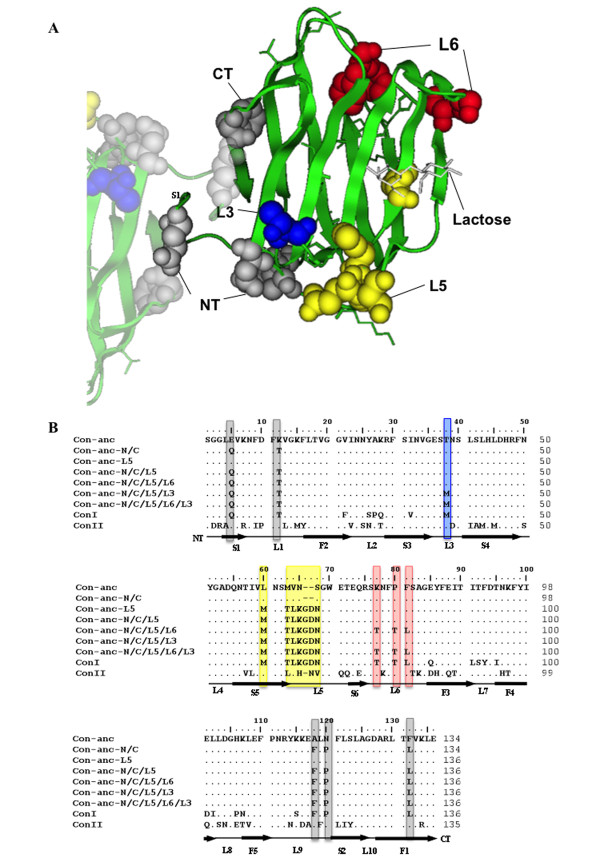
**Structure and mutants of Con-anc**. (A) 3D structure of Con-anc with liganded lactose, which was predicted by homology modeling, based on congerin I. The amino acid residues in N- and C-termini (NT and CT, respectively) and loops 3, 5, and 6 (L3, L5, and L6, respectively) of Con-anc mutants are represented by the space-filling model. (B) Aligned amino acid sequences of ConI, ConII, Con-anc, and Con-anc mutants. The sequences were aligned using the ClustalW program. Residue numbers of ConI were used as reference for all the congerins and mutants in this study. Positions of the strands (S1--S6, F1--F5) and loops (L1--L10) are indicated by thick and thin horizontal lines, respectively. The mutated amino acid positions are boxed in color.

**Figure 2:**
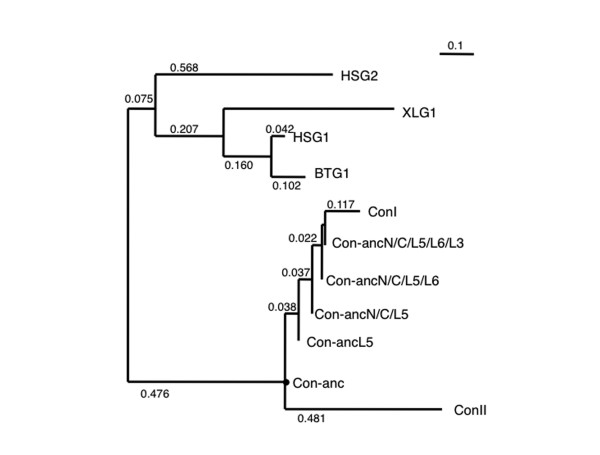
**The molecular phylogenetic tree of galectins including Con-anc and mutants**. The phylogenetic tree was inferred from the amino acid sequences.

As the N- and C-terminal regions located at the inter-subunit interface of the congerins and L5 region involved in the formation of the lactose-binding site are different for ConI and Con-anc (Figure 1A), first, the N- and C-termini and the L5 regions of Con-anc were mutagenized. Thus, the following 3 Con-anc mutants were prepared: (1) Con-anc-N/C, in which the N- and C-termini were substituted with the corresponding residues of ConI; (2) Con-anc-L5, in which the L5 region was substituted with the corresponding sequence of ConI; and (3) Con-anc-N/C/L5, which had mutations of both Con-anc-N/C and Con-anc-L5 (Figure 1B). As the binding ability of Con-anc-N/C/L5 was only 30%-40% of that of ConI (described later), the other structural elements responsible for the strong binding activity of ConI, namely, L6 and L3 (Thr38), located at the carbohydrate-binding cleft, were also investigated (Figure 1A). Thus, the Con-anc mutants, in which the L6 and L3 regions were substituted with the corresponding sequences of ConI, i.e., Con-anc-N/C/L5/L6, Con-anc-N/C/L5/L3, and Con-anc-N/C/L5/L6/L3 (Figure 1B), were prepared, and their carbohydrate-binding activities were determined by frontal affinity chromatography (FAC). To analyze the molecular evolutionary relationship among the ancestral mutants and ConI, the phylogenetic tree including the chimera mutants between Con-anc and ConI was constructed (Figure 2). The tree branched out from the node of each mutant with zero distance, suggesting that the chimera mutants are hypothetical ancestral mutants of ConI on molecular evolution.

### Thermostability of Con-anc and mutants

**Figure 3:**
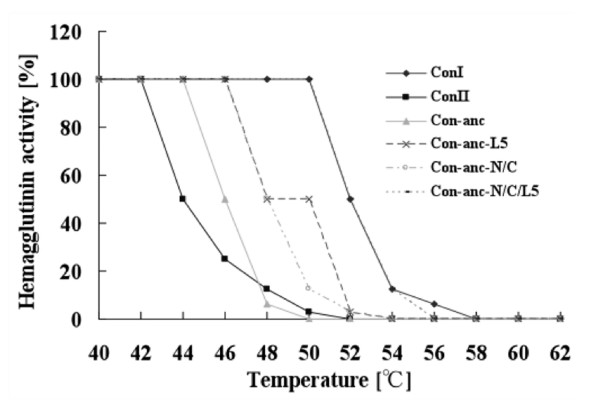
**Thermal stabilities of ConI, ConII, Con-anc, and Con-anc mutants**. The residual hemagglutination activities were measured after incubation at various temperatures for 30 min.

Figure 3 shows the thermostabilities of ConI, ConII, and Con-anc and its mutants. Interestingly, all mutants, i.e., Con-anc-N/C, Con-anc-L5, and Con-anc-N/C/L5, possessed higher thermostabilities than Con-anc; Con-anc-N/C/L5, in particular, showed a high stability, comparable with that of ConI. The half-activity retention temperature (T_m_)--the temperature at which 50% hemagglutination activity was retained after 30 min of incubation--of Con-anc and ConII were 44 and 46°C, respectively. On the other hand, Con-anc-N/C and Con-anc-L5 showed a ~2°C higher T_m _value (48°C) than that of Con-anc, and Con-anc-N/C/L5 showed a 6°C higher T_m _value (52°C) than that of Con-anc, but were comparable with that of ConI. These results indicate that the N- and C-terminal regions along with the L5 region of ConI are involved in conferring high thermostability, although the N- and C-terminal regions are located at the inter-subunit interface and are involved in the strand-swap structure of ConI. Substitutions of amino acid residues at the N- and C-termini, namely, E5Q, K12T, A118F, N120P, and F132L, may stabilize the strand-swap structure, making it comparable with that of ConI. The strand swapping (or domain swapping) is a motivity of quaternary structure formation in protein evolution; a protein becomes multimeric by donating a part (β strand) of the molecule to a cognate molecule and then accepting the corresponding portion from the cognate. The strand-swap in ConI is a variation of domain swapping, because the conformation of the swapped strands is changed from anti-parallel (in Con II) to parallel (in Con I). The strand-swap structure in ConI increases the inter-subunit contact surface area and the number of inter-subunit hydrogen bonds, resulting the enhancement of its dimeric stability and its cross-linking activity [19]. Thus, the N- and C-terminal regions contribute to the dimeric stability associated with the hemagglutinating activity, although it is not clear whether Con-anc adopts the strand-swap conformation or not. Meanwhile, substitution of the L5 region of Con-anc with the corresponding sequence of ConI conferred a more hydrophilic property to L5, as demonstrated by the hydropathy values of the L5 regions of Con-anc (LNSMVNS) and ConI (MNSTLKGDN), which were estimated to be 1.3 and -10.6, respectively [24]. In general, the hydrophilic residues on the surface of proteins are believed to increase thermostability [25-27].

### Carbohydrate-binding properties of Con-anc and mutants

**Figure 4:**
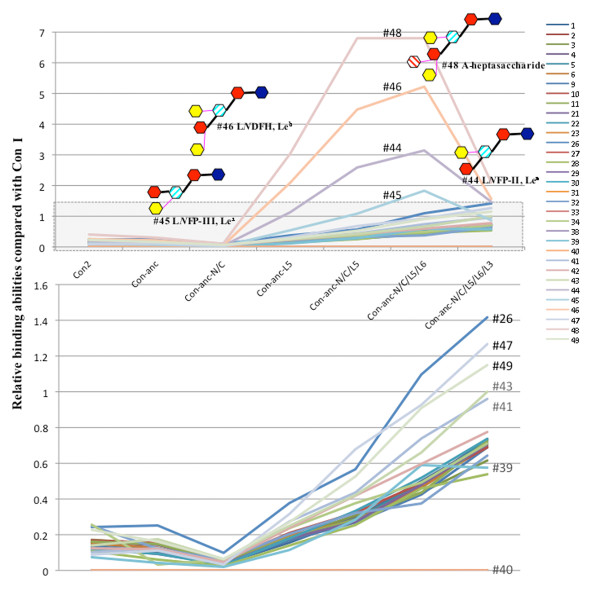
**The relative sugar-binding activities of Con II and Con-anc mutants when compared with that of Con I**. Scale for the PA sugars, except for L*N*FP-II, L*N*FP-III, L*N*DFH, and A-heptasaccharide (#44, #45, #46, and #48, respectively), was expanded. The PA sugar numbers are provided in Additional File 3: Supplemental Figure S1.

The carbohydrate-binding specificities and activities of ConI, ConII, and Con-anc and its mutants, namely, Con-anc-N/C, Con-anc-L5, Con-anc-N/C/L5, Con-anc-N/C/L5/L6, Con-anc-N/C/L5/L3, and Con-anc-N/C/L5/L6/L3, all of which were determined by FAC, are summarized in Table 1. In the previous report [23], we demonstrated that the sugar-binding specificity of Con-anc-L5 was similar to that of ConII, except for the specific oligosaccharides including lacto-*N*-biosyl (Galβ1-3GlcNAc) or lacto-*N*-neobiosyl (Galβ1-4GlcNAc) moieties, especially, lacto-*N*-fucopentaose-II (L*N*FP-II, Lewis a (Le^a^)) (#44), lacto-*N*-difucohexaose (L*N*DFH, Le^b^) (#46), and A-heptasaccharide (#48). However, in the present work, we have demonstrated increased carbohydrate-binding activity of Con-anc-N/C/L5 with respect to almost all sugars, when compared with those of Con-anc. Although the binding affinity of Con-anc-N/C to sugars decreased, the N- and C-terminal regions of ConI was found to increase the binding activity together with the introduction of the L5 sequence of ConI. Therefore, in terms of carbohydrate-binding activity, L5 should be the predominant structural element for high binding activity, and the N- and C-terminal regions may play an auxiliary role in carbohydrate binding by increasing the structural stability or slightly altering the structure. Con-anc-N/C/L5/L6 showed higher affinity toward almost all sugars than Con-anc-N/C/L5 (Table 1). Interestingly, the specific binding activity of Con-anc mutants, namely, Con-anc-L5, Con-ancN/C/L5, and Con-anc-N/C/L5/L6, were greatly increased against L*N*FP-II (#44), L*N*FP-III (#45), L*N*DFH (#46), and A-heptasaccharide (#48), all of which contained fucosyl-GlcNAc, by 20- to 30-fold when compared with the affinity of Con-anc, and by 3- to 7-fold when compared with that of ConI (Figure 4). These results suggest that the L5 and L6 regions of ConI may be involved in the high binding affinity to fucosyl-GlcNAc-containing sugars such as L*N*FP-II (#44), L*N*FP-III (#45), L*N*DFH (#46), and A-heptasaccharide (#48). On the other hand, the activity of Con-anc-N/C/L5/L6/L3 was reduced by approximately 30-50% of that of Con-anc-N/C/L5/L6 against these carbohydrates (Figure 4), although Con-anc-N/C/L5/L3 showed almost the same binding activity as Con-anc-N/C/L5 (data not shown). These results suggest that Thr38/Met38 residues in L3 cooperate with L6 to modulate the carbohydrate binding specificity.

**Table 1:** Comparison of the dissociation constants (*K*_d_) of ConI, ConII, Con-anc, and Con-anc mutants.

Sugar #	ConI			ConII			Con-anc			Con-anc-N/C			Con-anc-L5			Con-anc-N/C/L5		
	
	*K*_d _(μM)	**ratio to Con-anc**.	ratio to ConI	*K*_d _(μM)	**ratio to Con-anc**.	ratio to ConI	*K*_d _(μM)	**ratio to Con-anc**.	ratio to ConI	*K*_d _(μM)	**ratio to Con-anc**.	ratio to ConI	*K*_d _(μM)	**ratio to Con-anc**.	ratio to ConI	*K*_d _(μM)	**ratio to Con-anc**.	ratio to ConI
01	0.50	10	1	3.8	1.4	0.13	5.2	1	0.10	13	0.41	0.04	3.2	1.6	0.16	1.7	3.1	0.30
02	0.36	7	1	2.3	1.0	0.15	2.4	1	0.15	7.1	0.34	0.05	2.0	1.2	0.18	1.2	1.9	0.29
03	0.24	7	1	1.4	1.1	0.17	1.6	1	0.15	4.9	0.33	0.05	1.3	1.2	0.18	0.78	2.1	0.31
04	0.36	7	1	2.3	1.1	0.16	2.5	1	0.15	7.6	0.33	0.05	1.9	1.3	0.19	1.3	2.0	0.29
05	0.58	8	1	4.0	1.1	0.14	4.6	1	0.13	14	0.32	0.04	3.2	1.4	0.18	2.0	2.3	0.29
06	0.51	8	1	3.4	1.2	0.15	4.0	1	0.13	12	0.35	0.04	2.6	1.6	0.20	1.8	2.2	0.28
09	0.51	9	1	3.8	1.2	0.13	4.7	1	0.11	14	0.34	0.04	3.0	1.6	0.17	1.9	2.4	0.26
10	0.42	6	1	2.5	1.1	0.17	2.7	1	0.16	8.0	0.33	0.05	2.0	1.3	0.20	1.3	2.0	0.31
11	0.37	7	1	2.3	1.1	0.16	2.5	1	0.15	7.3	0.34	0.05	2.0	1.3	0.19	1.3	1.9	0.28
21	1.2	10	1	9.6	1.3	0.13	13	1	0.10	33	0.39	0.04	6.7	1.9	0.18	4.4	2.9	0.28
22	1.4	11	1	12	1.3	0.12	15	1	0.09	46	0.32	0.03	7.8	1.9	0.18	4.2	3.5	0.32
23	-	-	-	-	-	-	-	-	-	-	-	-	-	-	-	-	-	-
41	0.96	8	1	9.4	0.8	0.10	7.7	1	0.12	23	0.34	0.04	3.5	2.2	0.28	2.2	3.5	0.43
42	0.31	8	1	2.5	1.0	0.12	2.6	1	0.12	6.5	0.40	0.05	1.3	2.0	0.24	0.74	3.5	0.42
43	0.35	6	1	2.5	0.8	0.14	2.0	1	0.18	5.5	0.36	0.06	1.4	1.4	0.26	0.83	2.4	0.42

26	6.8	4	1	28	1.0	0.25	27	1	0.26	69	0.39	0.10	18	1.5	0.37	12	2.2	0.55
27	-	-	-	-	-	-	-	-	-	-	-	-	-	-	-	-	-	-
28	2.8	17	1	26	1.8	0.11	46	1	0.06	112	0.41	0.02	20	2.2	0.13	11	4.2	0.25
29	-	-	-	51	4.7	-	242	1	-	332	0.73	-	168	1.4	-	215	1.1	-
30	-	-	-	66	3.0	-	196	1	-	364	0.54	-	190	1.0	-	696	0.3	-
31	-	-	-	-	-	-	-	-	-	-	-	-	-	-	-	-	-	-
32	4.5	8	1	18	2.0	0.25	37	1	0.12	74	0.50	0.06	22	1.7	0.21	14	2.6	0.32
33	-	-	-	-	-	-	-	-	-	-	-	-	-	-	-	-	-	-
34	6.4	8	1	25	1.9	0.26	49	1	0.13	104	0.47	0.06	27	1.8	0.24	17	2.8	0.37
38	-	-	-	-	-	-	-	-	-	-	-	-	-	-	-	-	-	-
39	2.3	24	1	31	1.7	0.07	54	1	0.04	116	0.47	0.02	20	2.8	0.12	8.1	6.7	0.28
40	-	-	-	75	0.8	-	63	1	-	178	-	-	136	-	-	94	-	-
44	4.4	13	1	24	2.3	0.18	55	1	0.08	126	0.44	0.04	3.9	14	1.14	1.7	32	2.6
45	13	13	1	122	1.4	0.11	171	1	0.08	550	0.31	0.02	24	7.3	0.55	12	15	1.1
46	9.4	5	1	37	1.2	0.26	44	1	0.21	183	0.24	0.05	4.5	9.8	2.10	2.1	21	4.5
47	3.8	9	1	43	0.8	0.09	34	1	0.11	115	0.30	0.03	12	2.7	0.31	5.6	6.1	0.69
48	17	3	1	42	1.3	0.40	56	1	0.30	166	0.34	0.10	5.6	9.9	2.99	2.5	22	6.6
49	10	6	1	44	1.4	0.23	62	1	0.16	158	0.39	0.06	37	1.7	0.27	19	3.3	0.53
50	-	-	-	-	-	-	-	-	-	-	-	-	-	-	-	-	-	-

**Sugar #**	**Con-anc-N/C/L5/L6**			**Con-anc-N/C/L5/L6/L3**														
		
	***K*_d _(μM)**	**ratio to Con-anc**.	**ratio to ConI**	***K*_d _(μM)**	**ratio to Con-anc**.	**ratio to ConI**												
												
01	1.0	5.0	0.47	0.70	7.4	0.71												
02	0.77	3.1	0.46	0.49	4.9	0.73												
03	0.55	2.9	0.44	0.39	4.1	0.62												
04	0.75	3.3	0.48	0.49	5.1	0.74												
05	1.2	3.9	0.50	0.80	5.7	0.73												
06	1.1	3.6	0.46	0.73	5.5	0.70												
09	1.2	3.9	0.43	0.74	6.4	0.69												
10	0.86	3.1	0.48	0.61	4.4	0.68												
11	0.85	2.9	0.44	0.51	4.8	0.72												
21	2.5	5.0	0.49	1.7	7.5	0.73												
22	2.7	5.4	0.51	1.9	7.6	0.71												
23	-	-	-	-	-	-												
41	1.3	5.9	0.73	1.0	7.7	0.96												
42	0.52	4.9	0.59	0.40	6.4	0.76												
43	0.53	3.7	0.66	0.35	5.5	0.99												
26	6.2	4.3	1.1	4.8	5.5	1.42												
27	-	-	-	-	-	-												
28	6.1	7.6	0.46	5.2	8.8	0.53												
29	-	-	-	-	-	-												
30	-	-	-	-	-	-												
31	-	-	-	-	-	-												
32	12	3.1	0.38	7	5.6	0.68												
33	-	-	-	-	-	-												
34	13	3.7	0.48	9	5.7	0.75												
38	-	-	-	-	-	-												
39	3.9	14	0.58	4	15	0.64												
40	28	-	-	238	0.27	-												
44	1.4	39	3.1	3.0	19	1.49												
45	7.1	24	1.8	15	11	0.87												
46	1.8	24	5.1	6.0	7.3	1.57												
47	4.1	8.3	0.94	3.0	11	1.30												
48	2.5	22	6.7	8.1	6.9	2.08												
49	11	5.9	0.95	8.7	7.2	1.16												
50	-	-	-	-														

Ratios of *K*_a _(1/*K*_d_) values of congerins and their mutants to those of Con-anc and ConI were calculated. PA sugar numbers are provided in Additional File 3: Supplemental Figure S1.

Furthermore, the structural comparison of sugars, for which each mutant was either recognized specifically or not, showed that Con-anc-N/C/L5 and Con-anc-N/C/L5/L6 increased the recognizing specificity to an α1,4-fucosylated *N*-acetyl glucosamine (Lewis A, Le^a^) but not α1,3-fucosylated *N*-acetyl glucosamine (Lewis X, Le^x^) (Table 2). This indicates that ConI has evolved via accelerated evolution under significant selection pressure to acquire the binding activity to specific carbohydrates including α1,4-fucosylated *N*-acetyl glucosamine. It is known that the fucosylation occurs throughout nature and is concerned with the cell-cell interaction and cell migration in the physiological and pathological processes ranging from fertilisation and development through to pathological events and cell death [28,29]. In pathogenic bacterium, the fucosylated oligosaccharides have been found in *Helicobacter pylori*, which is a human pathogenic Gram-negative bacterium causing gastritis and gastric adenocarcinoma. Fucosylated antigens, Le^x ^and Le^y^, expressed on lipopolysaccharide of the microorganism play an important role in the infection, mimic host cell surface glycoconjugates and induce autoantibodies. Recently, fucose-specific lectins, F-type lectins, have been isolated from the serum from several fishes such as *Anguilla japonica *[30], *Anguilla anguilla *[31], *Morone saxatilis *[32], *Sparus aurata *[33], and *Dicentrarchus labrax *[34]. They have been proposed to play a role as molecular recognition factors in innate immunity. In the case for conger eel, F-type lectins have not yet been identified although C-type lectin and galectins have been isolated from serum and skin mucus [35,13,14]. These observations permit us to speculate that ConI may function as a surrogate of F-type lectin besides the function as galectin in conger eel.

**Table 2:** Comparison of the relative sugar binding activities of Con-anc mutants, Con-anc-N/C/L5, Con-anc-N/C/L5/L6, and Con-anc-N/C/L5/L6/L3.

Compared sugars	Ratio of relative binding activities			Different structure(s) between two compared sugars
		
	Con-anc N/C/L5	Con-anc N/C/L5/L6	Con-anc N/C/L5/L6/L3	
#45/#41	2.48	2.48	0.9	α1,3- fucosyl(GlcNAc)
#43/#42	1.01	1.11	1.29	α1,2- fucosyl(Gal)
#44/#42	6.18	5.27	1.89	**α1,4-fucosyl(GlcNAc)**
#46/#42	10.7	8.76	2.02	α1,2- fucosyl(Gal)/α1,4- fucosyl(GlcNAc)
#47/#42	1.62	1.55	1.63	α1,2- fucosyl(Gal)/α1,3- GalNAc(Gal)
#48/#42	16.2	11.4	2.71	**α1,4-fucosyl(GlcNAc)**/α1,2- fucosyl(Gal)/α1,3-GalNAc(Gal)
#46/#43	10.6	7.91	1.57	**α1,4-fucosyl(GlcNAc)**
#46/#44	1.73	1.66	1.07	α1,2- fucosyl(Gal)
#47/#43	1.61	1.4	1.27	α1,2- fucosyl(Gal)
#48/#43	16.1	10.3	2.1	**α1,4-fucosyl(GlcNAc)**/α1,3- GalNAc(Gal)
#48/#46	1.52	1.3	1.34	α1,3- GalNAc(Gal)
#48/#47	10	7.34	1.66	**α 1,4-fucosyl(GlcNAc)**

PA-sugar numbers are provided in Additional File 3: Supplemental Figure S1.

**Figure 5:**
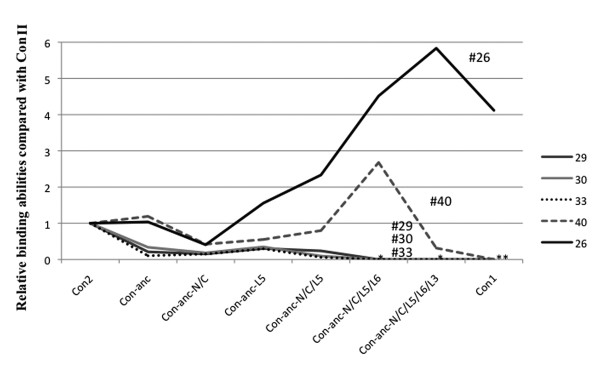
**The relative sugar-binding activities of Con I and Con-anc mutants when compared with that of Con II**. Asterisk (*) indicates no binding activities against sugars #29, #30, and #33, and asterisks (**) indicate no activities against #40 in addition to #29, #30, and #33. The PA sugar numbers are provided in Additional File 3: Supplemental Figure S1.

On the other hand, ConII showed a binding affinity to α2,3-sialyl galactose-containing sugars such as GM3 [NeuAc] (*N*-acetylneuraminic acid), GM3 [NeuGc] (*N*-glycolylneuraminic acid), and GD1a as described in a previous report [23], and ConI simply could not bind to GM3 and GD1a. Although Con-anc mutants, namely, Con-anc-N/C, Con-anc-L5, and Con-anc-N/C/L5, also showed binding affinities to α2,3-sialyl galactose-containing sugars--GM3 (NeuAc) (#29), GM3 (NeuGc) (#30), and GD1a (#33)--with very low interactions when compared with ConII, Con-anc-N/C/L5/L6 and Con-anc-N/C/L5/L6/L3 lost their binding activities against GM3 (NeuAc) (#29), GM3 (NeuGc) (#30), and GD1a (#33), indicating that the L5 and L6 structures of ConII contribute to the recognition of α2,3-sialyl galactose (Figure 5). The SPR analysis of Con-anc mutants using the GM3-immobilized sensor chip confirmed their binding affinity to α2,3-sialyl galactose-containing sugars (Table 3). Con-anc-N/C and Con-anc showed similar GM3-binding activity (*K*_d _= 4.9 × 10^-5 ^and 6.8 × 10^-5 ^M, respectively). On the other hand, Con-anc-L5 showed decreased affinity to GM3 (*K*_d _= 2.6 × 10^-4 ^M), and Con-anc-N/C/L5 showed negligible affinity to GM3 (*K*_d _= 1.0 × 10^-2 ^M). These observations indicate that the L5 and L6 regions of ConI are responsible for the strong binding affinities to α1,4-fucosylated *N*-acetyl glucosamine in exchange for the binding affinities to α2,3-sialyl galactose.

**Table 3:** Comparison of the dissociation constants (*K*_d_) of Con-anc, Con-anc-N/C, Con-anc-L5, and Con-anc-N/C/L5 to the immobilized GM3.

	*K*_d _(M)
Con-anc	6.8 × 10^-5^
Con-anc-N/C	4.9 × 10^-5^
Con-anc-L5	2.6 × 10^-4^
Con-anc-N/C/L5	1.0 × 10^-2^

### Molecular dynamics (MD) simulation

**Figure 6:**
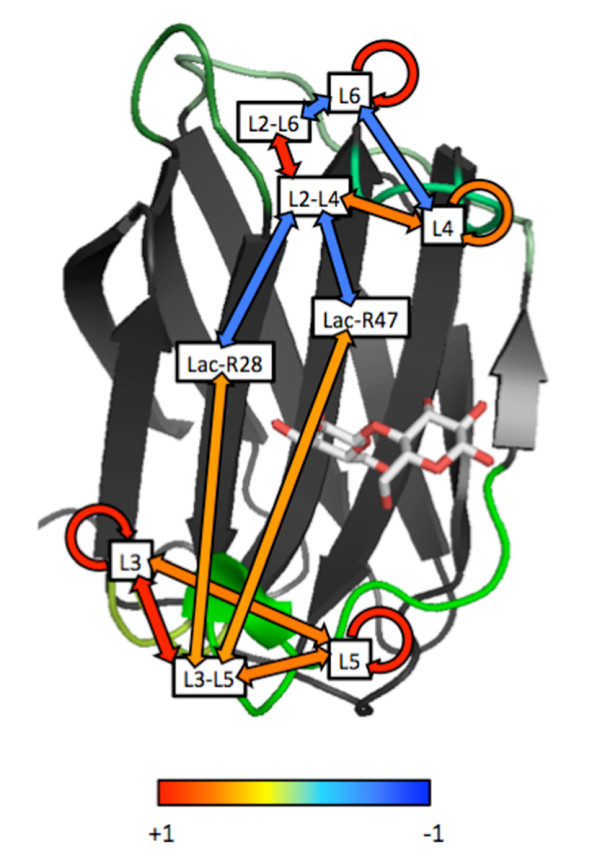
**Correlation of inter- and intra-loop hydrogen bond formation during MD simulation**. Correlation coefficients of hydrogen bond formation rates are shown on the structure of ConI. L3, L4, L5, and L6 represent the intra-loop bonds. L2--L4, L2--L6, and L3--L5 indicate the inter-loop bonds. Lac-R28 and Lac-R47 are the bonds between the residues and lactose. The colors of the arrows connecting the labels indicate the degree of correlation from positive (red) to negative (blue), as shown in the color bar.

L3 and L6 regions do not directly interact with the bound sugar in the crystal structure of ConI, although the mutants of these loops demonstrated significant alterations in the sugar-binding activity. To investigate the roles of these loops in sugar recognition, a 4-ns MD simulation of the dimeric ConI-lactose complex was performed. Cooperative behaviors within and between the loops and sugar-binding residues were evaluated as the correlation coefficients of the hydrogen bond formation rates during the simulation. As a result, high correlations within and between L3 and L5 were detected (Figure 6). Furthermore, the hydrogen bonds between L3 and L5 revealed a correlation with the bonds connecting Arg28 and Arg47 to lactose. On the other hand, L6 cooperated with L4 through the inter-loop connections mediated by L2, because L2-L6 and L2-L4 hydrogen bonds were highly correlated (Figure 6). This cooperation of loops, L6-L2-L4, showed a negative correlation with the lactose-binding hydrogen bonds, Lac-R28 and Lac-R47. These observations implied that the L6-L2-L4 and L3-L5 networks of the loops might have an antagonistic effect on sugar binding. As L4 and L5 were directly involved in sugar binding, these results suggested that the structural compatibility between loops L3-L5 and L6-L2-L4 might affect the sugar-binding activity, as observed in the Con-anc mutants.

### Apoptotic activities of Con-anc mutants

**Figure 7:**
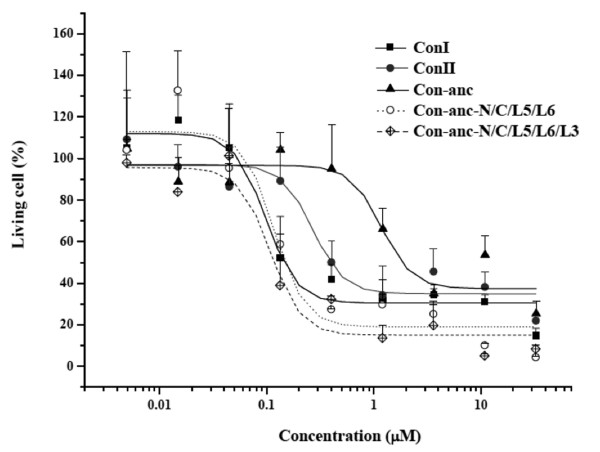
**Cytotoxic effects of ConI, ConII, and Con-anc mutants on Jurkat cells**. Solid square and continuous line represent ConI; solid circle and continuous line represent ConII; solid triangle and continuous line indicate Con-anc; hollow circle and dotted line indicate Con-anc-N/C/L5/L6; and hollow diamond and broken line represent Con-anc-N/C/L5/L6-T38 M. The calculated ED_50 _values (50% effective dose) were as follows: ConI, 0.098; ConII, 0.27; Con-anc, 1.2; Con-anc-N/C/L5/L6, 0.12; and Con-anc-N/C/L5/L6/L3, 0.11.

Figure 7 showed the dose-dependent cytotoxic effects of ConI, ConII, and Con-anc and its mutants on apoptotic activities for Jurkat cells. ConI showed a stronger apoptosis-inducing activity than ConII, similar to their carbohydrate-binding activities as described in the previous study, we found that ConI and ConII could strongly induce apoptosis in the human T-cell lines and Jurkat cells, and that their apoptotic activities are induced via lectin-carbohydrate interactions [36]. In the present study, the cytotoxic activities of Con-anc mutants were assayed using the Jurkat cells to evaluate the correlations between the apoptotic activities and carbohydrate-binding specificities of Con-anc and its mutants, in addition to the present-day congerins, ConI and ConII. The apoptotic activities of Con-anc and its mutants were positively correlated with their agglutinating activities, suggesting that Con-anc-N/C/L5/L6 and Con-anc-N/C/L5/L6/L3, whose carbohydrate-binding activities were almost the same (50-100%) as that of ConI, could produce apoptosis-inducing activities comparable with those of ConI. On the other hand, Con-anc, which had similar sugar-binding activity as that of ConII, showed a lower apoptosis-inducing activity than ConII (Figure 7).

Numerous galectins have been indicated as the functional molecules that control the fate of a cell [5,7,8,37,38]. In particular, there are many reports indicating galectins as apoptosis-inducing factors, e.g., galectin-1 [37], galectin-2 [39], galectin-3 (exogenous) [40], galectin-8 [41,42], and galectin-9 [43]. It is known that galectin-1-induced apoptosis requires its binding to the glycoproteins, including CD7, CD43, and CD45, on the surface of the T-cells [44-46]. These glycoproteins have some *O*-linked and/or *N*-linked glycans, and are modified by some glycosyltransferases via alteration of their susceptibility to galectin-1-induced apoptosis [47,48]. In addition, congerins also bind to CD7, CD43, and CD45. Neuraminidase-treated Jurkat cells were 3- and 5-fold more susceptible to ConI and ConII, respectively, than the non-treated Jurkat cells (unpublished data), indicating that the carbohydrate structure on the T-cell surface is important for the induction of apoptosis by congerins.

### Evolutionary process of congerins from ancestral gene

At the gene duplication event, the ancestral congerin Con-anc showed comparable thermostability and similar carbohydrate-binding specificities, with those of ConII, except for α2,3-sialyl galactose-containing sugars such as GM3 and GD1a. Thus, the gene encoding ConII has evolved in an accelerated manner from the ancestral gene to acquire the ability specific to pathogenic marine bacteria via the recognition of α2,3-sialyl galactose [20,23]. On the other hand, ConI has evolved from the ancestral congerin Con-anc to increase the binding activity against various sugars by modifying the N- and C-termini and L5, L6, and L3 regions. Particularly, modifying the L5 and L6 regions of Con-anc to ConI showed strong binding specificities against α1,4-fucosylated *N*-acetyl glucosamine. These findings emphasize that the carbohydrate-binding ability and the specificities of galectins can be controlled by modifying the loop structures.

**Figure 8:**
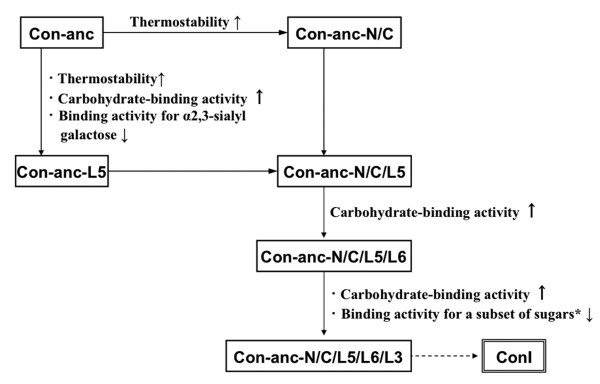
**Evolutionary pathway of ConI**. Each step in the pathway represents one functional evolutionary process. *A subset of sugars includes L*N*FP-II, L*N*DFH, and A-heptasaccharide (#44, #46, and #48, respectively).

In general, the rational designing of protein is a conventional and useful method to study the structure-function relationship of the protein with the partial molecular evolutionary information such as sequence alignments. However, it is difficult to predict and determine the effects of various mutations if several amino acids synergistically act as structural factors and exert multiple effects. In the present study, tracing analysis of molecular evolution of galectins by using ancestral gene and its mutated forms has enabled the more direct investigation of the structure-function relationship of proteins. In fact, we have elucidated the correlations between the molecular evolution (or amino acid substitutions) and functional diversification of ConI (Figure 8), which have revealed the detailed structural elements responsible for ligand specificity to L*N*FP-II, L*N*FP-III, L*N*DFH, and A-heptasaccharide (#44, #45, #46, and #48), respectively.

## Conclusions

The tracing analysis of molecular evolution, a protein engineering approach employing the reconstruction of probable ancestral forms based on phylogenetic trees and their mutants, is a powerful approach that not only reveals the molecular evolution process and determinants of selection pressures, but also helps to study the structure-function relationships of proteins.

## Methods

### Design and preparation of chimera mutants of Con-anc and ConI

The Con-anc mutants described in this study are summarized in Figure 1 and Additional File 1: Supplemental Table S1. These mutants were constructed by inverted polymerase chain reaction (PCR) amplification with some modifications [49], using PrimeSTAR HS DNA polymerase (TaKaRa Bio Inc., Japan) containing 2 ng of template DNA, 200 μM of each dNTP, and 0.3 μM of each primer. The pTV-Con-anc [23] and mutated pTV-Con-anc plasmids were used as templates, and oligonucleotide primers containing unique restriction enzyme and mutation sites were used for PCR (Additional File 2: Table S2). The reaction mixtures were cycled 30 times, with each cycle running at 98°C for 10 s, 55°C for 15 s, and 72°C for 3 min 30 s. The mutagenized PCR products were purified by agarose gel electrophoresis, using Wizard^® ^SV Gel and PCR Clean-Up System (Promega, USA), and subsequently digested with a unique restriction enzyme. The DNA fragment was self-ligated and then transformed into competent *Escherichia coli *JM109. The nucleotide sequences of the mutant plasmids were confirmed by DNA sequencing. Recombinant Con-anc mutants were prepared by a method previously reported for Con-anc [23]. In brief, each mutant was purified by affinity chromatography on an HCl-treated Sepharose 4B column (GE Healthcare, UK), followed by anion-exchange chromatography on a 5-ml HiTrap Q column (GE Healthcare). The purity of each mutant was confirmed by sodium dodecyl sulfate-polyacrylamide gel electrophoresis (SDS-PAGE). The phylogenetic tree of galectins including the chimera mutants between Con-anc and ConI was constructed by the maximum likelihood (ML) method in the PAML software package [50] using their amino acid sequences. Sequences data of galectins were retrieved from SwissProt databases. The entry codes for amino acid sequences are, respectively: ConI, leg1_conmy (p26788); ConII, leg2_conmy (q9yic2); BTG1 bovine galectin-1, leg1_bovin (p11116); HSG1 human galectin-1, leg1_human (p09382); HSG2 human galectin-2, leg2_humen (p05162); XLG1 Xenopus galectin-1, q98ud4.

### Thermostability measurements

Thermostabilities of Con-anc mutants were assessed by their residual hemagglutination activities using 2% rabbit erythrocytes plated on 96-well microtiter plates after incubation for 30 min at various temperatures ranging from 38 to 62°C in 50-mM Tris-HCl buffer (pH 7.5), followed by cooling on ice. The experiment was performed in duplicate, and separately repeated three times.

### Carbohydrate-binding properties

The carbohydrate-binding specificities and activities of Con-anc mutants were determined by frontal affinity chromatography (FAC) [51-53] in the same manner as that adopted for Con-anc [23]. The structures of the 34 kinds of pyridylaminated (PA) oligosaccharides used in FAC analysis are shown in Additional File 3: Supplemental Figure S1. In the SPR analysis, lyso-GM3 (Takara Bio Inc.) was immobilized on the sensor chip CM5 (GE Healthcare) via amino group, using carbodiimide chemistry, according to the manufacturer's manual.

### Molecular dynamics simulation

The coordinates of the ConI-lactose complex were retrieved from the Protein Data Bank (PDB code 1c1l), and the dimer structure was constructed on the basis of the biological unit matrix. Molecular dynamics (MD) simulation was performed for 4 ns using the SANDER module of AMBER 9 program suite. The AMBER03 force field [54] and GLYCAM 04 parameter set were used for the protein molecules and lactose, respectively. The system was solvated with TIP3P water molecules. To maintain the overall electrostatic neutrality conditions, Na^+ ^ions were added to the simulated systems. The initial unfavorable atomic contacts were removed by energy minimization with 1500 steps. The simulation was then started at 5 K, with the initial velocities adopted from a Maxwellian distribution, followed by heating from 5 to 300 K over 50 ps. Subsequently, a 100-ps equilibration was performed at 300 K. Electrostatic interactions were calculated without distance cutoff by using the particle-mesh Ewald method [55]. The SHAKE algorithm was applied to constrain the bond lengths with hydrogen atoms [56]. The MD trajectories were analyzed using the PTRAJ module of AMBER. The correlation coefficients of cooperative hydrogen-bond formation were evaluated to detect the relationships between the loops (L2, L3, L4, L5, and L6) and sugar-binding sites (Arg28 and Arg47). The first 3-ns trajectory was divided into 200-ps (20 steps) bins, and the formation rates within the time-bins were calculated for each hydrogen bond that showed a 10-90% overall formation rate, excluding the transiently or permanently formed ones. The formation rate is the fraction of the snapshot structures that have the objective hydrogen bond in the total structures within the time-bin. The correlation coefficients for the pairs of hydrogen bonds were evaluated from the arrays of the formation rates. The correlation coefficient can be defined as

where, *x*_*i *_and *y*_*i *_are the formation rates of the hydrogen bonds *x *and *y *within the time bin *i*, respectively. The coefficients were averaged within and between the loops or sugar-binding residues.

### Cell culture and in vitro cell assays of Con-anc and its mutants

The cytotoxic activities of Con-anc and its mutants were assessed by using Jurkat cells [57,58] as the target cells. The Jurkat cells were maintained in a RPMI-1640 medium supplemented with 10% fetal bovine serum and 1% antibiotic-antimycotic solution at 37°C in 5% CO_2 _atmosphere. The Jurkat cells were grown in 96-well microtiter plates for the assay. Three-fold serial dilutions of Con-anc or its mutants were added to the confluent cells. After culturing for 24 h at 37°C in 5% CO_2 _atmosphere, the cell viability was evaluated using the cell proliferation reagent WST-1 (Dojindo, Japan), according to the manufacturer's instructions. Subsequently, the chromophores of WST-1 were measured by absorbance at 450 nm. The assay was performed in triplicate, and confirmed separately three times to assess the reproducibility.

## Authors' contributions

TO, AKo, TS and KM designed research. AKo, SY and TO performed molecular and biochemical experiments. TS and AKi performed molecular dynamics simulation and bioinformatics studies. TO, AKo and TS wrote the paper.

## Supplementary Material

Additional file 1 **Table S1 - Con-anc mutants in this report**. * For example, E5Q indicates that Glu5 of Con-anc substituted to corresponding ConI residue, Gln. Click here for file

Additional file 2 **Table S2 - Primer list in this report**. * Upper is sense primer and lower is antisense primer in each row. The substitution sites were underlined, and unique restriction enzyme sites were in italics. Click here for file

Additional file 3 **Figure S1 - Schematic representation of PA oligosaccharides used in FAC analysis**. 01-23, *N*-linked glycans; 26-50, glycolipid glycans. The reducing terminal sugar of each carbohydrate was pyridylaminated. All PA oligosaccharides were purchased from TaKaRa Bio (Kyoto, Japan). The numbers assigned to them are based on their product numbers. Each vertex of a hexagon indicates the position of the anomeric carbons in each monosaccharide. Thin pink and thick black lines represent α and β bonds, respectively. Glc, glucose; Gal, galactose; Man, mannose; Fuc, fucose; GlcNAc, *N*-acetylglucosamine; GalNAc, *N*-acetylgalactosamine; NeuAc, *N*-acetylneuraminic acid; and NeuGc, *N*-glycolylneuraminic acid. Click here for file
