# Supplementary figures and images for: Protein engineering of conger eel galectins by tracing of molecular evolution using probable ancestral mutants
Source: BMC Evol Biol. 2010 Feb 14;10:43. doi: 10.1186/1471-2148-10-43 (PMC2843614; doi:10.1186/1471-2148-10-43)

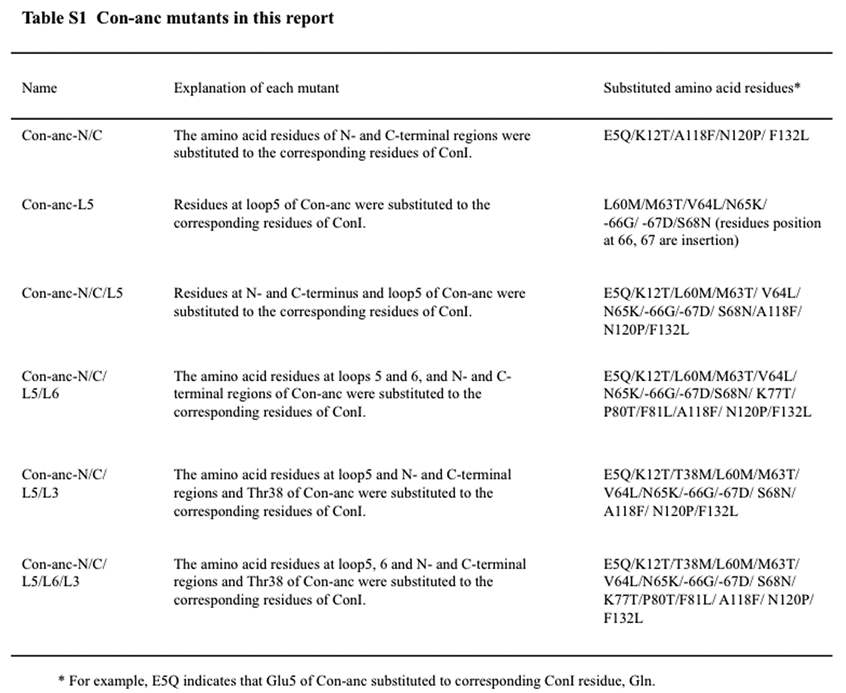

Supplement: Additional file 1 — Table S1 - Con-anc mutants in this report. * For example, E5Q indicates that Glu5 of Con-anc substituted to corresponding ConI residue, Gln. [file 1471-2148-10-43-S1.TIFF]

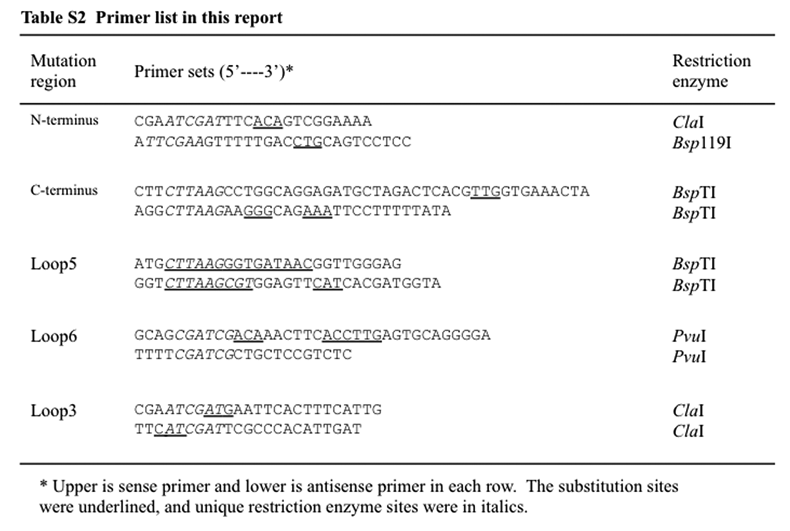

Supplement: Additional file 2 — Table S2 - Primer list in this report. * Upper is sense primer and lower is antisense primer in each row. The substitution sites were underlined, and unique restriction enzyme sites were in italics. [file 1471-2148-10-43-S2.TIFF]

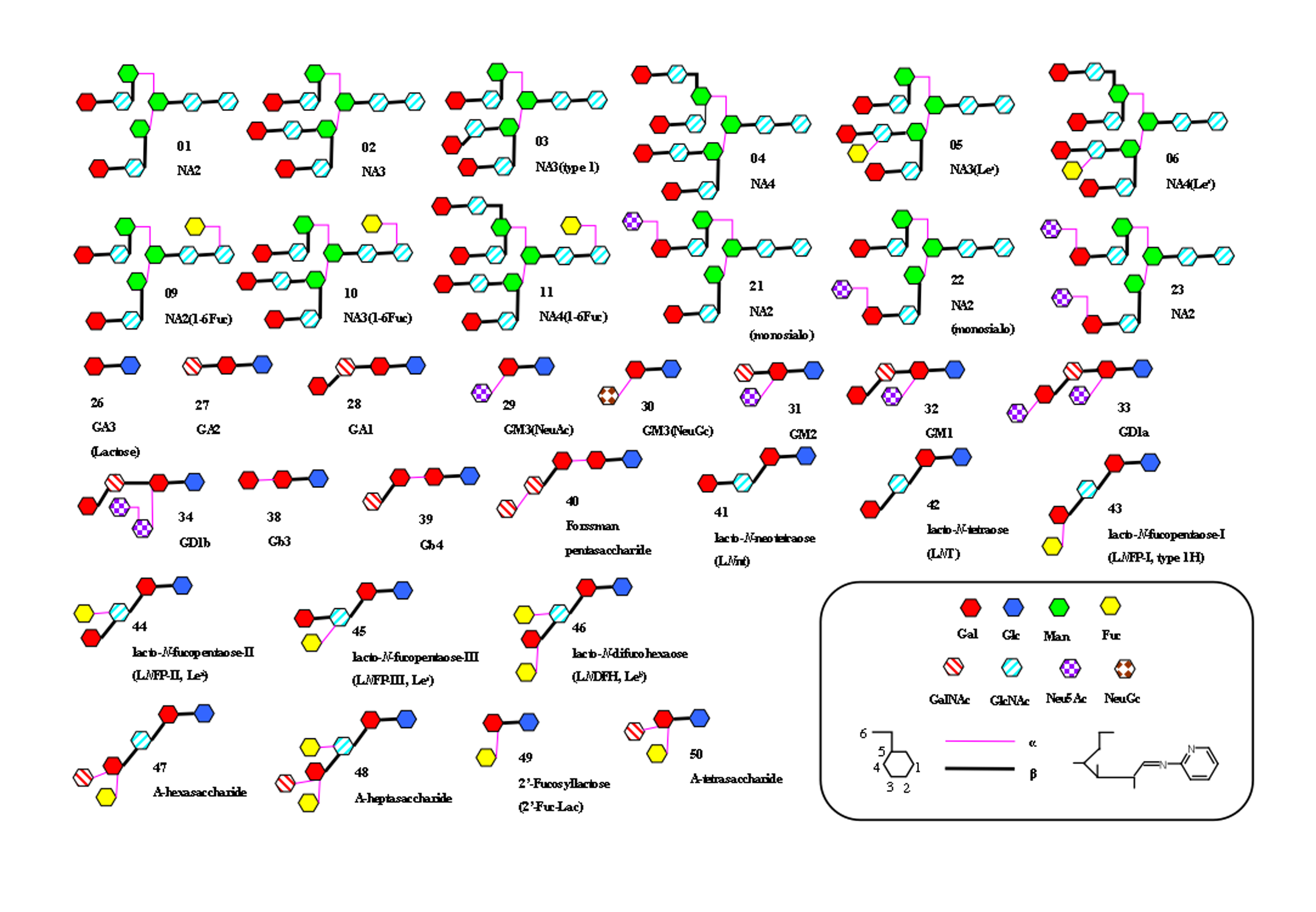

Supplement: Additional file 3 — Figure S1 - Schematic representation of PA oligosaccharides used in FAC analysis. 01-23, N-linked glycans; 26-50, glycolipid glycans. The reducing terminal sugar of each carbohydrate was pyridylaminated. All PA oligosaccharides were purchased from TaKaRa Bio (Kyoto, Japan). The numbers assigned to them are based on their product numbers. Each vertex of a hexagon indicates the position of the anomeric carbons in each monosaccharide. Thin pink and thick black lines represent α and β bonds, respectively. Glc, glucose; Gal, galactose; Man, mannose; Fuc, fucose; GlcNAc, N-acetylglucosamine; GalNAc, N-acetylgalactosamine; NeuAc, N-acetylneuraminic acid; and NeuGc, N-glycolylneuraminic acid. [file 1471-2148-10-43-S3.TIFF]
